# Collagen Scaffolds in Veterinary Medicine: Safety, Sustainability, and Clinical Accessibility of Mammalian- and Marine-Derived Sources—A Scoping Review

**DOI:** 10.3390/ani16162529

**Published:** 2026-08-13

**Authors:** Anna Carolo, Roberta Sacchetto, Marco Patruno

**Affiliations:** Department of Comparative Biomedicine and Food Science, University of Padova, Viale dell’Università 16, Legnaro, 35020 Padova, Italy; anna.carolo@unipd.it (A.C.); marco.pat@unipd.it (M.P.)

**Keywords:** regenerative veterinary medicine, biomaterials, mammalian collagen, marine collagen

## Abstract

Collagen-based biomaterials are widely used in regenerative medicine, particularly in human clinical practice for musculoskeletal and dermal applications. In veterinary medicine, however, their adoption remains limited, mainly due to high costs, limited species-specific evidence, and the lack of standardized clinical protocols. Additionally, the need for extensive purification and traceability to meet regulatory safety requirements significantly impacts production costs and scalability. Marine-derived collagen (MDC), obtained from vertebrate (e.g., fish) and invertebrate (e.g., jellyfish and sponges) sources, has recently emerged as a promising alternative to mammalian-derived collagen. MDC can be sourced from seafood industry by-products, offering a more sustainable and potentially cost-effective solution. These materials present reduced biological risks, including negligible zoonotic transmission and absence of prion contamination, along with tunable structural and functional properties suitable for scaffold and hydrogel development. This scoping review, conducted in accordance with the PRISMA-ScR guidelines, provides a comparative analysis of mammalian- and marine-derived collagen-based scaffolds, focusing on their relevance for veterinary applications based on 61 sources selected from a systematic search of the PubMed and Scopus databases. Biological safety, functional performance, environmental sustainability, and economic accessibility are critically discussed. Particular attention is given to the potential of MDC to improve the accessibility, affordability, and sustainability of regenerative therapies in veterinary medicine.

## Simple Summary

Collagen-based biomaterials are widely used in regenerative medicine, but their application in veterinary practice remains limited due to high costs, limited species-specific evidence, and a lack of standardized protocols. This scoping review, conducted following the PRISMA-ScR guidelines, critically compares mammalian- and marine-derived collagen-based biomaterials for veterinary regenerative medicine, evaluating their biological performance, safety, sustainability, and economic accessibility. A systematic search of the PubMed and Scopus databases was performed, and 61 sources were selected following title/abstract and full-text screening. Studies investigating collagen-based scaffolds for regenerative medicine involving veterinary species or relevant animal models were included, while cosmetic, food, and industrial applications were excluded. The selected literature was qualitatively synthesized into thematic sections addressing structural properties, safety, functional performance, sustainability, and translational potential. Mammalian collagen offers superior mechanical strength and thermal stability, making it preferable for load-bearing applications such as bone and tendon repair. Marine collagen provides advantages in terms of safety, sustainability, and availability, making it attractive for soft tissue and minimally invasive applications. Treating-dispensation and processing strategies frequently influence biomaterial performance more than collagen source alone. Therefore, an application-driven approach to biomaterial selection is recommended, and improved species-specific research and standardization are essential to expand regenerative therapies in veterinary medicine.

## 1. Introduction

Collagen-based biomaterials represent one of the most extensively investigated and clinically adopted platforms in regenerative medicine. As the main structural protein of the extracellular matrix (ECM), collagen plays a pivotal role in maintaining tissue architecture and regulating cellular behavior, including adhesion, proliferation, and differentiation [1]. These properties have led to its widespread application in tissue engineering strategies targeting skin, bone, cartilage, and periodontal regeneration. In particular, collagen scaffolds and hydrogels are considered a gold standard due to their intrinsic biocompatibility, biodegradability, and ability to mimic native tissue environments [2].

Traditionally, collagen used in biomedical applications is primarily derived from mammalian sources, especially bovine and porcine tissues. Although these materials have been extensively used in regenerative medicine, their broader application is associated with several limitations. Concerns related to zoonotic disease transmission, including prion-associated risks such as bovine spongiform encephalopathy, as well as immunogenic reactions, ethical considerations, and religious constraints, have raised questions about long-term safety and acceptance [3,4,5]. Additionally, the production and processing of mammalian collagen are associated with relatively high costs and supply chain dependencies, which may limit accessibility, particularly outside high-resource clinical settings [6,7].

In parallel, increasing attention has been directed toward alternative collagen sources, particularly marine-derived collagen obtained from fish skin, scales, bones, and other seafood processing by-products. Marine-derived collagen has emerged as a promising alternative owing to its wide availability, lower risk of transmitting mammalian pathogens, and its potential contribution to circular economy strategies through the valorization of seafood industry waste [8,9]. These characteristics have stimulated growing interest in its application as a sustainable biomaterial for regenerative medicine.

Despite these advantages, marine collagen presents intrinsic limitations, particularly in terms of mechanical performance, which may restrict its direct use in mechanically demanding applications. Consequently, processing strategies such as crosslinking, composite formulation, and scaffold engineering are often required to improve its functional performance [2,10,11]. A detailed comparison of the physicochemical and biological properties of mammalian- and marine-derived collagen is presented in the following sections.

These considerations become particularly relevant in the context of veterinary regenerative medicine. Although significant advances have been made in the development of regenerative therapies for animal health, their clinical adoption remains limited. Factors such as high treatment costs (that limit accessibility to a narrow patient population), lack of standardized protocols, and restricted access to advanced biomaterials have contributed to a translational gap between experimental research and routine veterinary practice [6,12,13]. In this framework, the selection of biomaterials is not only a technical decision but also an economic and practical one. Materials that are sustainable, cost-effective, and scalable may offer a more realistic pathway toward widespread clinical implementation, especially in veterinary settings where resource constraints are more pronounced.

Given the multidimensional nature of the research question—encompassing biological performance, safety, sustainability, and economic accessibility across heterogeneous study designs, outcome measures, and clinical contexts—a scoping review approach was selected as the most appropriate methodology. Unlike systematic reviews, which are designed to address focused clinical questions amenable to a single PICO (Population, Intervention, Comparison, Outcome) framework, scoping reviews are specifically suited to map the available evidence across broad and complex topics, identify knowledge gaps, and provide decision-making frameworks [14]. The present review addresses a research question that spans multiple dimensions of biomaterial science and veterinary clinical practice, which cannot be reduced to a single answerable PICO question, and for which the available evidence is heterogeneous in terms of study design, animal models, and outcome measures. A scoping review methodology therefore represents the most appropriate and scientifically defensible approach to synthesize this body of evidence.

Based on these premises, this review aims to address the following question: how do mammalian- and marine-derived collagen biomaterials compare in terms of biological performance, safety, sustainability, and economic accessibility, and how can these differences inform fit-for-purpose selection in veterinary regenerative medicine?

The objective of this scoping review is to critically compare mammalian- and marine-derived collagen-based biomaterials for veterinary regenerative medicine, highlighting how biomaterial selection should be tailored to the biological, mechanical, and clinical requirements of each application.

## 2. Methods

This scoping review was conducted in accordance with the PRISMA Extension for Scoping Reviews (PRISMA-ScR) guidelines [14], with the aim of providing a comprehensive overview of collagen-based scaffolds in veterinary regenerative medicine, with particular emphasis on comparisons between mammalian- and marine-derived sources.

### 2.1. Search Strategy

A systematic literature search was performed using two electronic databases, PubMed and Scopus. The search was initially conducted in June 2025 and subsequently verified on 25 July 2025. No lower date limit was applied, and all records published up to the date of submission were considered. The search was restricted to articles published in English.

A systematic search was conducted in PubMed as follows:

(“collagen scaffold*” OR “collagen biomaterial*” OR “marine collagen” OR “mammalian collagen” OR “fish collagen”)
AND
(“veterinary” OR “canine” OR “equine” OR “feline” OR “ovine” OR “animal model”)
AND
(“tissue engineering” OR “regenerative medicine” OR “wound healing” OR “bone regeneration” OR “tendon repair” OR “scaffold”)
and Scopus (using equivalent search terms in title, abstract, and keywords).

### 2.2. Study Selection

The database search identified 182 records from PubMed and 368 from Scopus, yielding a total of 550 records. After removal of 185 duplicates, 365 unique records remained for title and abstract screening.

Studies were included if they:

- Investigated collagen-based biomaterials or scaffolds;
- Addressed applications in regenerative medicine or tissue engineering;
- Involved veterinary species or animal models relevant to translational veterinary research;
- Reported biological, mechanical, or translational outcomes;
- Provided physicochemical, structural, or methodological data directly relevant to the comparative evaluation of collagen sources.

Title and abstract screening was conducted using the following exclusion criteria:

- Applications involving organ systems outside the scope of this review (e.g., nervous, cardiac, or gastrointestinal systems);
- Cosmetic or food industry applications;
- Industrial or non-biomedical uses;
- Conference proceedings or abstracts lacking full data;
- Corrigenda;
- Studies focused exclusively on biochemical characterization without direct relevance to the comparative evaluation of collagen sources for regenerative applications.

Following this stage, 245 records were excluded, leaving 120 articles for full-text assessment.

Full-text articles were evaluated for relevance to the objectives of the review, particularly regarding the comparison between mammalian- and marine-derived collagen biomaterials. An additional 59 records were excluded at this stage due to insufficient relevance or lack of data on collagen-based biomaterials in regenerative applications.

A total of 61 studies were ultimately included in the scoping review.

**Figure 1:**
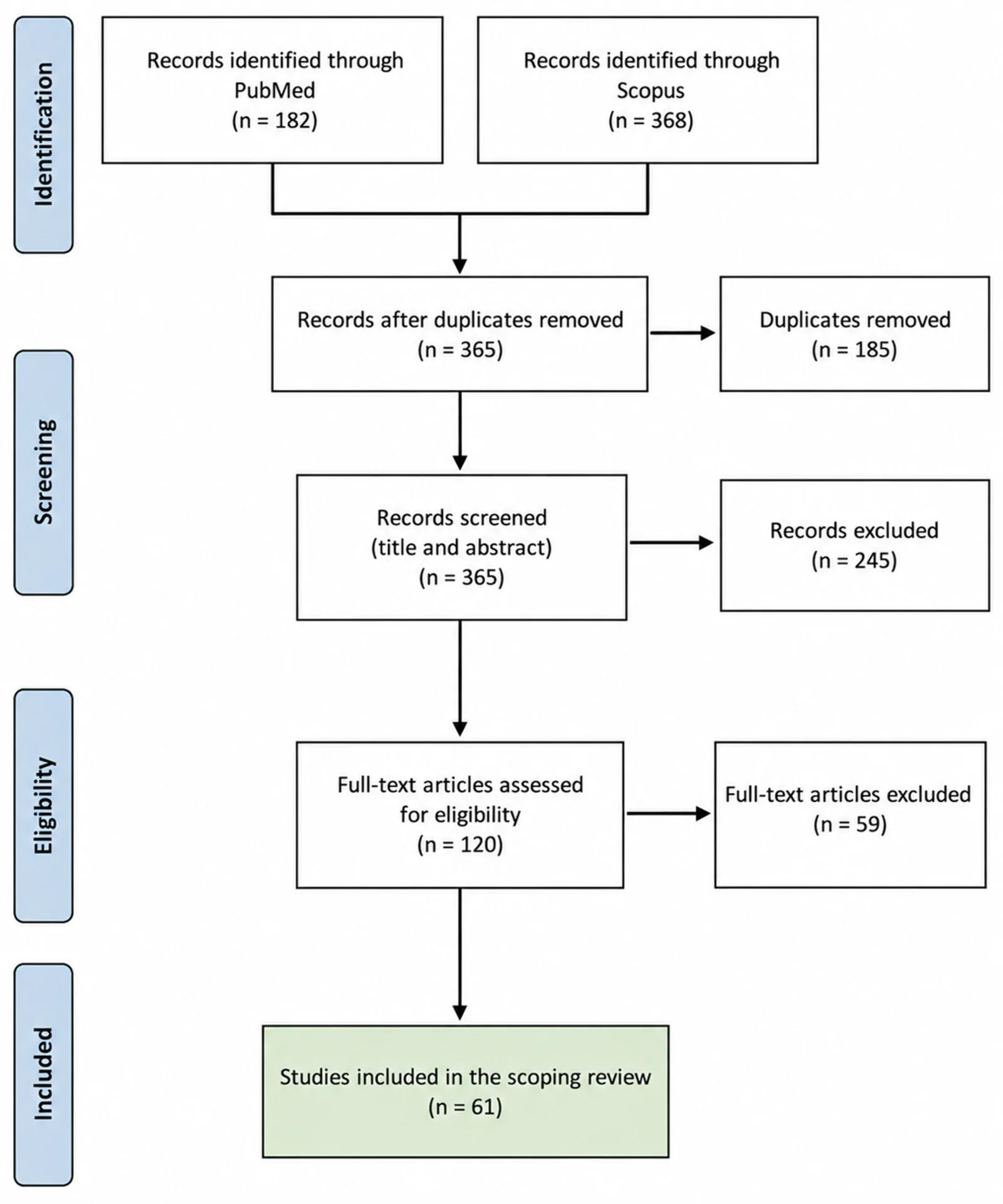
PRISMA-ScR flow diagram illustrating the study selection process for the scoping review. A total of 182 records were identified from PubMed and 368 from Scopus; after removal of 185 duplicates, 365 records were screened by title and abstract. Following full-text assessment of 120 articles, 61 studies were included in the final review.

The study selection process is summarized in the PRISMA-ScR flow diagram (Figure 1).

### 2.3. Data Charting Process

Data were charted by a single reviewer (A.C.) using a standardized extraction framework in order to ensure consistency in data recording. All decisions regarding inclusion criteria, variables to be extracted, and interpretation of the charted data were agreed upon by all authors prior to screening and validated collectively throughout the process. Borderline cases identified during title/abstract and full-text screening were resolved through collegial discussion involving all co-authors, ensuring methodological consistency and reproducibility of the selection process. This approach is consistent with PRISMA-ScR guidelines for scoping reviews.

For each included source, the following information was recorded: author(s), year of publication, study design, collagen source (mammalian or marine), scaffold format, target tissue or application, animal model or veterinary species involved, key outcomes reported (biological, mechanical, or translational), and main conclusions relevant to the review objectives.

An overview of the studies included in the review is provided in Supplementary Material Table S1. Studies are categorized according to study design, collagen source, scaffold format, target application, and animal model/species. Principal outcomes are summarized together with their relevance to the comparison between mammalian and marine collagen.

### 2.4. Data Items

The following variables were sought from each included source: (i) collagen source and species of origin; (ii) scaffold or biomaterial format (e.g., sponge, hydrogel, membrane, or electrospun matrix); (iii) crosslinking or processing strategy applied; (iv) target tissue or clinical application; (v) animal model or veterinary species; (vi) biological outcomes (e.g., cell viability, proliferation, and differentiation); (vii) mechanical properties reported; (viii) in vivo regenerative outcomes where available; and (ix) sustainability or safety considerations. Where data were not explicitly reported, this was noted as not available.

### 2.5. Data Extraction and Synthesis

The selected literature was qualitatively synthesized and organized into thematic sections, including biological and structural properties, safety, functional performance, sustainability, and translational potential in veterinary clinical practice.

A completed PRISMA-ScR checklist is provided as Supplementary Material.

## 3. Overview of Mammalian- and Marine-Derived Collagen

Collagen is the most abundant structural protein in the extracellular matrix of vertebrates, organized as a right-handed triple helix composed of three polypeptide α-chains with a repeating Gly-X-Y sequence, where X and Y positions are frequently occupied by proline (Pro) and hydroxyproline (Hyp). The amino acid content at these positions (Pro + Hyp) is the primary determinant of triple-helix thermal stability and supramolecular assembly behavior [15]. At the supramolecular level, collagen molecules self-assemble hierarchically into microfibrils, fibrils, and fibers through fibrillogenesis, conferring the mechanical versatility characteristic of collagen-based tissues [7,16]. These structural properties are strongly source-dependent, as differences in Pro + Hyp content, fibril diameter, and denaturation temperature between mammalian and marine collagen translate directly into distinct mechanical, degradation, and biological profiles, as discussed in the following sections.

Mammalian collagen represents the most established source of collagen for biomedical and regenerative applications, being primarily obtained from bovine and porcine skin, tendon, and bone tissues [7,15]. Owing to well-established extraction procedures and extensive biomedical use, mammalian collagen currently represents the reference material for many scaffold-based regenerative applications [2,17,18].

Extraction methods for mammalian collagen, including acid solubilization and enzymatic processing, have been optimized to preserve triple-helix integrity while enabling large-scale production [15,18]. This has contributed to a relatively high degree of standardization across batches, which is a critical requirement for clinical translation and regulatory approval. These factors, combined with production costs and sourcing dependencies, highlight the need for alternative collagen sources that can complement or replace mammalian-derived materials in specific contexts.

In this context, marine-derived collagen has emerged as a promising alternative, particularly in response to safety and sustainability concerns associated with mammalian sources. MDC is primarily obtained from fish skin, scales, and bones, but also from invertebrate organisms such as jellyfish and marine sponges. A key advantage of marine collagen lies in its origin from seafood processing by-products, which enables the valorization of large volumes of otherwise discarded biomass within a circular economy framework [6,8].

Processing strategies, including crosslinking and scaffold fabrication, can substantially modify the physicochemical and biological properties of collagen-based biomaterials. These approaches are particularly relevant for marine collagen, where intrinsic limitations can be partially compensated through material engineering.

Collagen can be further processed into a variety of scaffold formats, including hydrogels, sponges, and fibrous matrices, each tailored to specific regenerative applications. Hydrogels, for example, provide highly hydrated and biomimetic environments suitable for cell encapsulation and soft tissue repair, while more structured scaffolds can support load-bearing functions. In addition, composite systems combining collagen with synthetic polymers, ceramics, or bioactive molecules have been widely explored to improve mechanical performance and functional versatility [11].

Overall, processing and scaffold fabrication act as a functional bridge between raw material properties and clinical requirements. By tuning structural and mechanical characteristics, these strategies enable a more flexible use of both mammalian and marine collagen, setting the stage for a comparative evaluation of their performance across different regenerative contexts.

## 4. Comparative Analysis of Collagen-Based Biomaterials

Collagen-based biomaterials share a conserved triple-helix architecture. Functionally relevant differences between collagen sources arise primarily from variations in amino acid content (Pro + Hyp), which govern triple-helix stability, fibrillogenesis, and enzymatic susceptibility. These differences are strongly species-dependent, with cold-water marine organisms typically exhibiting lower proline and hydroxyproline content compared to warm-water species and terrestrial mammals, resulting in reduced triple-helix stability and altered supramolecular organization [15,19,20,21].

As a consequence, mammalian collagen exhibits higher thermal stability, with denaturation temperatures typically in the range of 37–40 °C, supporting structural integrity under physiological conditions [15,20].

In contrast, marine collagen generally displays lower denaturation temperatures (~15–30 °C depending on species) [22,23,24], resulting in decreased molecular stability and increased conformational flexibility [21,25,26,27]. Although stabilization strategies such as crosslinking and composite formulation can improve its functional performance under physiological conditions, these approaches are discussed in more detail below.

These differences extend to fibrillogenesis, with mammalian collagen forming densely packed fibrils with larger diameters (~80–150 nm), whereas marine-derived collagen typically forms thinner and less organized fibrils (~30–100 nm), reflecting reduced intermolecular cohesion and structural heterogeneity [16,21,28,29,30].

Consequently, processing strategies such as crosslinking and composite scaffold design are often employed to improve the structural stability of marine-derived collagen under physiological conditions.

A critical and often underexplored limitation emerges when considering the application of marine-derived collagen in veterinary species. Unlike human physiology (~37 °C), the body temperature of most target veterinary patients—including dogs, cats, and horses—ranges between 38.0 and 39.5 °C. This creates a substantial thermal mismatch when using marine collagen with denaturation temperatures frequently below 30 °C, particularly for collagen derived from cold-water species. Under such conditions, non-stabilized marine collagen may undergo rapid conformational loss, transitioning toward gelatin-like structures with compromised mechanical integrity and increased susceptibility to enzymatic degradation [10,21,25].

This limitation is not merely theoretical and may represent an important translational challenge for veterinary applications. As a consequence, the successful application of marine collagen in veterinary contexts often requires appropriate stabilization strategies. Crosslinking approaches—chemical, physical, and enzymatic—as well as composite systems with synthetic polymers or inorganic phases are required to increase thermal resistance and maintain structural integrity under physiological conditions [17,27,31]. However, these interventions introduce additional challenges, including potential cytotoxicity, altered degradation kinetics, and increased manufacturing complexity.

Overall, these structural and physicochemical differences establish a fundamental trade-off between stability, processability, and downstream functional performance, which directly underpins scaffold design for regenerative applications.

## 5. Structure–Function Relationships and Biological Performance

### 5.1. Mechanical Properties and Load-Bearing Performance

These molecular and supramolecular distinctions translate directly into mechanical behavior. Mammalian collagen is generally associated with higher tensile strength and elastic modulus, making it suitable for load-bearing or semi-load-bearing applications such as bone, tendon, and periodontal scaffolds [15,16]. Marine-derived collagen exhibits lower stiffness and reduced resistance to mechanical stress, although these properties vary considerably depending on the source species and processing strategy [6,21]. However, all of these features together may be advantageous for soft tissue applications such as skin, cornea, or wound healing matrices [32].

### 5.2. Enzymatic Stability and Degradation Behavior

Collagen is primarily degraded via enzymatic pathways mediated by matrix metalloproteinases and collagenases, which cleave the triple-helix structure at specific accessible sites [7,33].

In mammalian collagen, the dense packing and hierarchical organization of fibrils create steric constraints that limit enzyme diffusion and partially restrict access to cleavage sites, resulting in more controlled degradation kinetics. In contrast, the lower packing density and reduced structural order of marine collagen enhance solvent penetration and facilitate enzyme diffusion, increasing enzyme accessibility to cleavage sites [16,17].

This structural openness, combined with lower thermal stability, promotes partial molecular unfolding and greater exposure of enzymatically labile regions, ultimately leading to faster degradation rates under physiological conditions [6,17,20,27]. Although often considered a limitation, this faster turnover can be advantageous in transient scaffolds or applications requiring rapid remodeling and integration.

### 5.3. Cell Adhesion, Proliferation, and Differentiation

Collagen acts as a bioactive extracellular matrix component that regulates cell fate through biochemical and biophysical cues. Cell–material interactions are mediated through integrin-mediated signaling and matrix-dependent mechanotransduction, which activate signaling pathways controlling adhesion, proliferation, and differentiation [34,35]. The outcomes are strongly influenced by scaffold composition, stiffness, architecture, and crosslinking state [21,32,34,35].

Mammalian collagen provides highly conserved binding motifs that support strong and reproducible cell adhesion, sustained proliferation, and well-characterized differentiation outcomes [7]. Marine-derived collagen also supports robust cytocompatibility and adhesion, although differences in sequence homology and structural organization may influence receptor affinity and downstream signaling intensity, particularly in differentiation pathways [6,20].

### 5.4. Scaffold Architecture and Application-Driven Design

Importantly, these biological responses are strongly modulated by scaffold architecture. Porous sponge-like constructs promote rapid cell infiltration and vascularization, hydrogels enable homogeneous cell encapsulation and diffusion-driven signaling, and fibrous or electrospun scaffolds provide contact guidance and anisotropic cell alignment. These structural formats significantly influence adhesion–proliferation–differentiation cascades as scaffold porosity and pore size directly regulate cell infiltration, nutrient diffusion, and tissue integration, often exceeding the influence of collagen source alone [11,21,32].

Processing strategies, including crosslinking, sterilization, and composite formulation, play a major role in determining scaffold performance by simultaneously influencing mechanical properties, degradation kinetics, and cell responses. In many cases, these modifications may have a greater impact on biological performance than the collagen source itself [11,17,21,27].

From a veterinary perspective, these differences acquire additional relevance when mapped onto specific clinical scenarios. For instance, equine tendon injuries or canine orthopedic defects require scaffolds capable of maintaining mechanical integrity under dynamic loading conditions, favoring highly stabilized mammalian collagen or reinforced composites [16,32]. Conversely, applications such as wound healing, dermal regeneration, or corneal repair may benefit from the faster remodeling and higher compliance of marine-derived scaffolds, provided that adequate thermal stabilization is achieved [6,10]. The scarcity of standardized in vivo veterinary studies remains a major limitation, highlighting the need for more species-specific validation.

According to recent in vivo studies, appropriately engineered marine-derived collagen scaffolds support this design-dependent paradigm even further, demonstrating that marine-derived collagen scaffolds, particularly when combined with inorganic phases such as hydroxyapatite, can enhance regenerative outcomes. For example, increased bone formation compared to bovine collagen-based controls has been reported in rabbit models, together with improved cellular infiltration and reduced inflammatory response [21].

The key physicochemical and biological differences between mammalian- and marine-derived collagen discussed above are summarized in Table 1, providing a comparative framework to guide application-driven biomaterial selection.

A detailed study-level comparison of collagen structural and physiochemical properties is available in the review by Yuan et al. 2026 [21], which readers are encouraged to consult for in-depth methodological comparisons.

## 6. Application-Specific Design Strategies in Veterinary Regenerative Medicine

The design of collagen-based biomaterials in veterinary regenerative medicine must be driven by tissue-specific functional requirements and supported by in vivo evidence whenever available. Differences between mammalian and marine collagen, particularly in terms of thermal stability, fibrillar organization, degradation kinetics, and optical properties, translate into distinct performance profiles across clinical applications [6,7,15]. Increasingly, comparative in vivo studies are beginning to clarify how these differences impact regenerative outcomes in veterinary-relevant models.

### 6.1. Load-Bearing Tissues (Bone, Tendon, and Ligament)

Musculoskeletal injuries represent a major clinical challenge in veterinary medicine, particularly in high-performance species such as sport horses, where pathologies such as superficial digital flexor tendon injuries are characterized by high recurrence rates, and in dogs, where critical-sized bone defects and non-unions remain difficult to manage clinically [38,39]. For these highly demanding mechanical environments, mammalian collagen remains the benchmark material due to its higher thermal stability and mechanical integrity, which are critical for maintaining scaffold architecture and supporting cellular load during the early, crucial phases of healing [37].

Recent in vivo work by Diogo et al. [40] directly compared marine- and mammalian-derived collagen scaffolds in a rabbit bone defect model, demonstrating that although mammalian collagen exhibited superior initial mechanical stability, marine collagen scaffolds supported comparable bone regeneration when combined with targeted crosslinking and mineral phases. Crucially, marine collagen displayed faster remodeling dynamics, a feature that may be highly advantageous in specific clinical scenarios where rapid biomaterial turnover and replacement by host neo-tissue are desired.

This context-dependent performance of marine collagen has been further enhanced through composite approaches. Marine-derived collagen scaffolds, when successfully reinforced with inorganic phases such as hydroxyapatite, significantly improved tissue regeneration outcomes in rabbit models. The authors reported an increased bone formation rate (~17.9% vs. ~12.9% in bovine controls), alongside enhanced cellular infiltration and a reduced host inflammatory response [40]. This integration of collagen with synthetic polymers or inorganic ceramics optimizes osteoconductivity and durability [20,32,41,42], reinforcing the concept that material selection alone is insufficient for hard tissues without an appropriate multi-scale composite design.

However, the direct translation of these findings to veterinary patients remains limited. Most available evidence derives from rabbit models, which do not fully reproduce the biomechanical and biological conditions encountered in clinical veterinary patients, particularly in highly loaded tissues such as equine tendons. Therefore, further in vivo studies in target veterinary species are needed before these approaches can be translated into routine clinical practice.

### 6.2. Soft Tissues (Skin, Cornea, and Wound Healing)

Soft tissue injuries and ophthalmic pathologies are among the most frequent clinical presentations in small animal practice. These include severe traumatic skin wounds, chronic pressure ulcers in geriatric or recumbent dogs, and ophthalmic conditions such as indolent corneal ulcers in brachycephalic breeds and feline corneal sequestra, all of which frequently result in delayed healing, secondary infections, and compromised tissue function [43].

Unlike hard tissues, soft tissue regeneration prioritizes mechanical compliance, primarily requiring biomaterials that support rapid cell infiltration and tissue remodeling. In this context, marine-derived collagen has attracted considerable interest because of its favorable biological properties and promising regenerative potential [6,13]. Several in vivo studies support this functional profile. In small animals in pre-clinical settings, marine collagen-based scaffolds significantly enhanced wound closure and granulation tissue formation in rat full-thickness skin defects, promoting superior collagen deposition and neovascularization [44]. Concurrently, accelerated re-epithelialization and improved dermal organization were reported in rodent models treated with marine collagen matrices [45].

Importantly, these rodent-based screening findings are consistent with targeted veterinary data. In a clinically relevant canine skin wound model, marine-derived collagen scaffolds promoted significantly faster epithelialization and excellent integration into the host tissue [46]. Additionally, a prototype skin substitute made of recycled marine-derived collagen significantly improved skin regeneration in a sheep wound model, further supporting the translational relevance of these biomaterials in large animal systems [47].

The convergence of rodent, ovine and canine evidence suggests that the faster enzymatic turnover of marine collagen directly drives accelerated healing dynamics. Furthermore, providing direct evidence in a canine model significantly bridges the translational gap, confirming the clinical safety and efficacy of marine-derived biomaterials in real veterinary patients.

Severe traumatic injuries and deep wounds often compromise not only skin integrity but also that of deep skeletal muscle. The intrinsic regenerative capacity of this tissue is sometimes insufficient to counteract the pathological remodeling triggered by extensive injuries. The damaged tissue is frequently replaced by fibrotic scar tissue, resulting in significant impairment of muscle function. New methods and materials, promoting skeletal muscle repair and function, are constantly being investigated. Mammalian collagen-based biomaterials are considered ideal candidates for engineering skeletal muscle tissue [48]. A recent study has demonstrated that sea urchin-derived collagen matrix represents a promising scaffold in this field [49]. These collagen-based models, mimicking the muscle extracellular matrix, support C2C12 cells cellularization and infiltration, thereby promoting progression of myoblast towards myogenic commitment [49,50].

In ophthalmic applications, scaffold transparency and precise fibrillar organization are the ultimate determinants of functional success, as the corneal stroma requires a highly ordered architecture to maintain optical clarity. Marine collagen has emerged as a promising alternative for corneal substitutes due to its lower fibril density and favorable light transmission properties.

Marine-derived collagen has been shown to be a promising material for corneal scaffolds, providing high transparency and supporting strong corneal epithelial cell growth both in vitro and in vivo. Additional studies have likewise demonstrated that collagen matrices from marine sources can promote corneal regeneration, offering good tissue biocompatibility and maintaining acceptable transparency in animal models [51,52].

While the most clinically advanced corneal implants have traditionally relied on mammalian or recombinant human collagen [53], marine-derived collagen represents a highly promising and cost-effective alternative for veterinary ophthalmology. In veterinary ophthalmology, marine collagen scaffolds could represent a promising alternative for the treatment of deep corneal ulcers or feline corneal sequestra. Their high transparency and favorable biological properties may help preserve optical function while reducing the need for more invasive surgical procedures. However, clinical evidence in veterinary patients remains limited and further validation is required.

### 6.3. Injectable and Minimally Invasive Systems

Injectable collagen-based systems are increasingly explored in veterinary medicine as minimally invasive therapeutic approaches, particularly in conditions such as osteoarthritis in dogs, periodontal disease in small breeds, and focal soft tissue defects where surgical intervention may be limited or contraindicated [54,55]. These systems are typically based on collagen hydrogels or sol–gel formulations that can be delivered in situ and undergo controlled gelation under physiological conditions.

From a materials perspective, injectable systems can be broadly classified into:

- Physically gelled hydrogels (temperature- or pH-induced fibrillogenesis);
- Chemically crosslinked hydrogels (e.g., EDC/NHS and genipin);
- Composite injectable systems incorporating cells, growth factors, or nanoparticles.

Marine-derived collagen may be particularly suitable for injectable application systems because of its relatively high solubility, lower viscosity, and favorable gelation behavior. These properties facilitate handling and enable minimally invasive delivery through standard syringes. In contrast, mammalian collagen often requires tighter control of temperature and pH to achieve reproducible fibrillogenesis, which can complicate clinical use [6,32].

Clinically relevant veterinary applications include:

- Wound filling and management of irregular traumatic defects in dogs and cats: Injectable collagen hydrogels can conform to irregular defects, serving as a provisional matrix promoting cell infiltration and tissue regeneration.
- Cartilage and joint applications: Collagen-based injectable systems have been explored for intra-articular delivery, often in combination with mesenchymal stem cells (MSCs), to support cartilage repair and modulate inflammation.
- Periodontal and oral regeneration: Injectable collagen systems may also provide a minimally invasive approach for periodontal regeneration by allowing direct delivery of biomaterials into periodontal defects [56,57,58].
- Drug and cell delivery platforms: Collagen hydrogels can serve as carriers for bioactive molecules (e.g., growth factors and antimicrobials) or cells, enabling localized and sustained therapeutic effects.

From a functional standpoint, the faster degradation of marine collagen can be advantageous in injectable systems, where rapid remodeling and integration are desired. However, this same property requires appropriate tuning of crosslinking density to prevent premature loss of structural integrity.

Despite these advantages, in vivo evidence specific to veterinary applications remains limited. Most studies focus on proof-of-concept or small animal models, and there is a lack of standardized protocols for formulation and delivery. Moreover, variability in gelation kinetics and mechanical properties can impact reproducibility and clinical translation.

Future development of injectable collagen systems will likely focus on improving control over gelation behavior, enhancing mechanical stability without compromising injectability, and integrating bioactive components to create multifunctional therapeutic platforms tailored to specific veterinary indications. Nevertheless, the lack of standardized veterinary-specific protocols and the variability in gelation behavior remain key barriers to routine clinical adoption.

### 6.4. Composite and Hybrid Systems

Composite and hybrid scaffolds have emerged as an effective strategy to tailor the biological and mechanical properties of collagen-based biomaterials for specific regenerative applications [37].

Several in vivo studies have shown that composite collagen-based scaffolds improve bone regeneration and structural stability compared with collagen alone, particularly when reinforced with hydroxyapatite or other inorganic phases [37,42].

In periodontal regeneration, studies such as Kosen et al. [56] and Ward et al. [58] in canine models have shown that collagen-based scaffolds can support the regeneration of complex soft–hard tissue interfaces, including alveolar bone and periodontal ligament. While these studies primarily use mammalian collagen, they highlight the importance of scaffold architecture and bioactivity over source alone, suggesting that appropriately engineered marine collagen systems could achieve comparable outcomes.

Overall, these findings support the continued development of hybrid collagen-based biomaterials for veterinary applications, consistent with the design principles discussed in Section 5.4. However, comparative studies directly evaluating mammalian- and marine-derived composite scaffolds in clinically relevant veterinary models are still limited.

Although composite systems offer clear functional advantages, their increased manufacturing complexity and the need for standardized fabrication protocols remain important challenges for clinical translation.

### 6.5. Translational and Veterinary-Specific Challenges

Despite promising results, several challenges continue to limit the clinical translation of collagen-based biomaterials in veterinary medicine. One of the main challenges is the limited availability of data demonstrating the long-term performance of collagen-based biomaterials under clinically relevant veterinary conditions, particularly in mechanically demanding tissues [6,40,46].

Another important limitation is the limited availability of in vivo data, especially in large animal and veterinary-specific models. While studies such as Diogo et al. [40] and Morales et al. [46] represent important advances, the overall body of evidence remains fragmented and insufficient for definitive clinical translation. This limits the possibility of establishing evidence-based recommendations for routine veterinary use.

Standardization is also a key concern. Marine collagen sources exhibit significant variability depending on species, extraction methods, and environmental factors, which can affect reproducibility and scalability [13].

Finally, the regulatory landscape for veterinary biomaterials remains less defined than in human medicine, potentially accelerating adoption but also increasing the risk of variability in product quality and performance.

Addressing these challenges will require additional standardized in vivo validation, improved material standardization, and the development of application-specific design guidelines grounded in both materials science and veterinary clinical needs, as further discussed in Section 7.

The application-specific design strategies discussed in this section, including tissue requirements, preferred collagen source, scaffold engineering approaches, and clinical decision criteria, are summarized in Table 2, providing a practical reference framework for collagen-based biomaterial selection in veterinary regenerative medicine.

**Figure 2:**
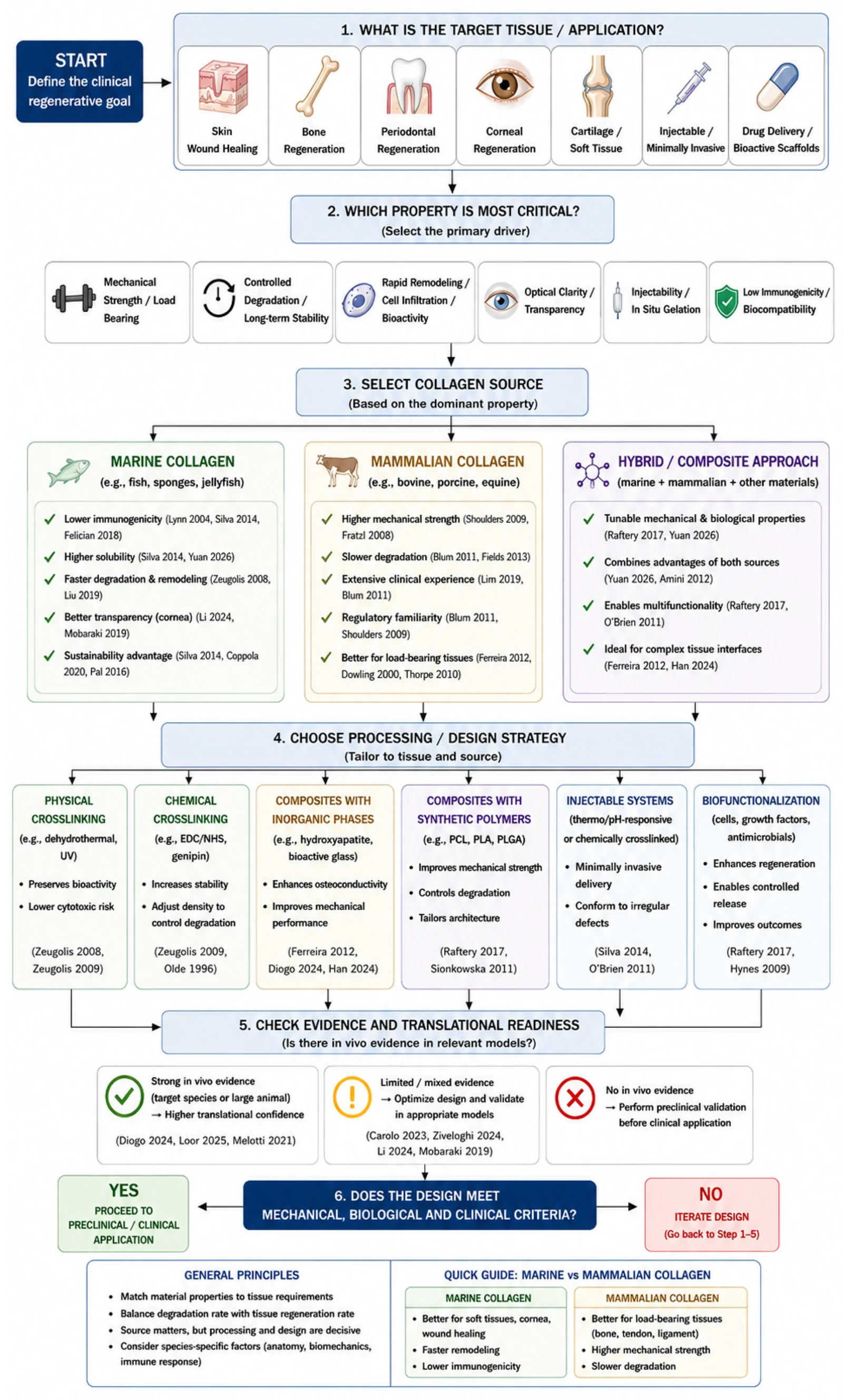
Application-driven decision framework for collagen source selection and scaffold design in veterinary regenerative medicine. The flowchart guides biomaterial selection through six sequential steps: definition of the target tissue, identification of critical functional properties, collagen source selection, processing strategy, evaluation of translational evidence, and final design validation. Reference citations embedded at each decision node indicate the specific studies supporting each proposed decision pathway, as discussed in detail in Section 6 [2,5,6,7,8,11,13,15,16,17,18,20,21,25,31,32,33,34,37,38,39,40,41,42,44,45,46,47,51,52,59].

To facilitate the clinical translation of these concepts, the application-driven decision framework described throughout this section is summarized in Figure 2, providing a practical stepwise guide for collagen source selection and scaffold design tailored to specific veterinary regenerative applications.

## 7. Challenges and Future Perspectives

Despite significant advances in collagen-based biomaterials for regenerative applications, several critical challenges continue to limit their full clinical translation, particularly in veterinary medicine.

### 7.1. Standardization and Reproducibility

One of the primary limitations across both mammalian and marine collagen sources is the lack of standardized extraction, purification, and processing protocols [60]. Variability in collagen source and processing protocols may substantially affect scaffold composition, mechanical properties and biological performance, limiting reproducibility across studies.

Future efforts should prioritize the development of standardized protocols and internationally accepted characterization criteria, including biochemical, mechanical, and ultrastructural benchmarks.

### 7.2. Mechanical Limitations and Structural Optimization

Although collagen-based biomaterials closely mimic the extracellular matrix, further optimization is still required to achieve adequate mechanical performance in load-bearing applications. To address these limitations, current research has focused on advanced crosslinking strategies and composite design. Chemical (e.g., EDC/NHS), enzymatic, and physical crosslinking approaches have been extensively explored to improve stability and mechanical performance [4,18]. In parallel, the incorporation of inorganic phases such as hydroxyapatite or bioactive glass, as well as synthetic polymers, has shown significant potential in enhancing stiffness and durability [32,37]. Future developments will likely rely on multi-scale design strategies enabling independent tuning of structural and functional properties.

### 7.3. Immunogenicity and Biosafety

While collagen is generally considered biocompatible, immunogenic responses remain a concern, particularly for mammalian-derived materials due to residual antigenic components and potential pathogen transmission. These risks, along with ethical considerations, have driven increasing interest in marine-derived collagen, which is associated with lower immunogenicity and reduced zoonotic risk [5,6,13].

However, despite these advantages, long-term in vivo validation of marine collagen remains limited, particularly in large animal models. A deeper understanding of host immune responses, degradation by-products, and long-term safety is still required. Advances in purification, decellularization, and molecular characterization will be critical to ensure biosafety and reproducibility.

### 7.4. Translational Gap and In Vivo Validation

One of the major challenges in the field is the persistent gap between promising in vitro results and robust in vivo evidence. While numerous studies have demonstrated efficacy in small animal models, relatively few have progressed to large animal models or clinically relevant veterinary applications. This limitation is particularly evident for marine collagen, which, despite encouraging preliminary data, lacks extensive translational validation compared to mammalian collagen, particularly in load-bearing and high-temperature physiological environments, where scaffold stability and degradation must be tightly controlled [6,7,46,47].

In particular, quantitative data correlating crosslinking density with in situ thermal stability and gelatinization rates in target veterinary species are currently lacking and represent a priority for future investigation.

The need for standardized and clinically relevant in vivo models has been emphasized in the broader biomaterials field [32,61]. Future research should prioritize comparative studies between collagen sources under controlled conditions, as well as long-term functional and histological outcomes in large animal models.

### 7.5. Regulatory and Manufacturing Challenges

Regulatory approval represents a significant hurdle, particularly for emerging biomaterials such as marine-derived collagen. Mammalian collagen products benefit from a longer clinical history and more clearly defined regulatory pathways, whereas marine collagen faces uncertainties related to classification, safety validation, and batch consistency.

In addition, scalability and cost-effectiveness remain critical barriers. Large-scale production with consistent quality is still challenging [59]. In fact, the economic advantage of marine collagen derived from seafood industry by-products must be critically re-evaluated in the context of medical-grade production. Although the raw material is inexpensive, extensive purification processes are required to remove contaminants such as heavy metals, lipids, odor-causing compounds, allergens, and pyrogens. When combined with Good Manufacturing Practice (GMP) requirements, these processes can significantly increase production costs, potentially offsetting the initial economic benefit compared to mammalian collagen sourced from controlled supply chains.

### 7.6. Emerging Trends and Future Directions

The field is rapidly evolving toward more sophisticated and multifunctional collagen-based systems. Emerging trends include hybrid biomaterials combining marine and mammalian collagen, advanced biofunctionalization strategies incorporating growth factors and bioactive molecules, and the development of smart materials capable of responding to environmental stimuli.

In addition, 3D bioprinting technologies are enabling precise spatial control over scaffold architecture, opening new possibilities for personalized regenerative approaches [62]. These strategies are particularly relevant in veterinary medicine, where species-specific variability presents additional challenges.

A major limitation across the field is that most available evidence derives from induced defects in small animal models, which do not fully replicate the complexity of spontaneous diseases in veterinary patients; therefore, future research should prioritize clinically relevant studies in dogs, cats, and horses. Ultimately, future advances will rely on application-driven biomaterial design supported by standardized methodologies and robust preclinical and clinical evidence.

Finally, it should be acknowledged that a systematic mapping of commercially available collagen-based biomaterials for veterinary applications, including information on collagen source, regulatory status, intended use, and approximate cost, was lacking and would represent a valuable contribution to the field. Therefore, the present dedicated scoping review, focused specifically on the commercial landscape of veterinary biomaterials, might serve as a practical complement to the evidence-based framework proposed in the present work, and is identified here as a priority direction for future research.

## 8. Conclusions

Collagen-based biomaterials represent a well-established and versatile platform for regenerative medicine, with growing relevance in veterinary clinical practice. The comparative analysis presented in this review highlights that neither mammalian nor marine-derived collagen can be considered universally superior. Rather, the selection of collagen source should be driven by the specific biological, mechanical, and clinical requirements of each application. Mammalian collagen remains the predominant choice for load-bearing and mechanically demanding tissues, where its higher thermal stability and structural integrity are critical. Marine-derived collagen, conversely, offers distinct advantages in soft tissue, wound healing, and minimally invasive applications, where its favorable remodeling dynamics, compliance, and sustainability profile represent meaningful clinical benefits. As discussed in Section 5.4, processing strategies often exert a greater influence on final biomaterial performance than collagen source alone, further supporting an application-driven rather than source-driven approach to material selection.

Despite these advances, significant challenges remain. As discussed in Section 7.4, direct validation in clinically relevant veterinary species remains limited and represents a priority for future research. Standardization of extraction, processing, and characterization protocols is still needed to ensure reproducibility and scalability. Future research should prioritize species-specific in vivo studies, comparative trials between collagen sources under controlled conditions, and the development of evidence-based design guidelines tailored to veterinary clinical needs. Addressing these gaps, also with the help of the present review, will be essential to bridge the translational mismatch between experimental biomaterials research and routine veterinary practice, ultimately expanding access to effective and sustainable regenerative therapies across animal species.

The methodological limitations of this scoping review should be acknowledged. The literature search was conducted using two electronic databases, PubMed and Scopus, which provide comprehensive coverage of the biomedical and biomaterial science literature relevant to the objectives of this review. The exclusion of additional databases such as CAB Abstracts or Web of Science represents a methodological limitation that may have resulted in the omission of some records indexed exclusively in veterinary-specific or multidisciplinary sources. However, the broad and inclusive search strategy adopted, combined with the comprehensive coverage of Scopus in life and materials sciences, is considered adequate to support the mapping objectives of this scoping review. Title and abstract screening and full-text assessment were performed collaboratively by all authors, ensuring cross-validation of inclusion decisions. Data charting was conducted by the first author using a standardized extraction framework to ensure internal consistency in data recording. No formal quality assessment of the included sources was performed, as this is not a requirement of the scoping review methodology; however, this limits the ability to draw definitive conclusions regarding the strength of the available evidence. Finally, the heterogeneity of study designs, animal models, and outcome measures across the included sources precluded quantitative synthesis and limited the comparability of findings across applications.

## Figures and Tables

**Table 1:** Comparative overview of structural, mechanical, degradation, and biological properties of mammalian- and marine-derived collagen-based biomaterials and their design-relevant implications.

Dimension	Parameter	Mammalian Collagen	Marine Collagen	Design-Relevant Implication	Key Reference
Structural and molecular	Amino acid content (Pro + Hyp, mol%)	~19–22 mol%	~12–18 mol% (cold-water fish); up to ~18–20 mol% (warm-water fish)	Governs triple-helix stability and fibrillogenesis	[15,20,25]
	Denaturation temperature (Td)	37–41 °C (bovine/porcine type I collagen)	15–20 °C (cold-water fish); 25–30 °C (warm-water fish)	Critical thermal mismatch in veterinary applications	[20,25,26]
	Fibril diameter	50–500 nm (tissue-dependent)	20–200 nm, more heterogeneous	Affects porosity, stiffness, cell infiltration	[15,16]
Mechanical	Young’s modulus (hydrated scaffolds, un-crosslinked)	~0.5–5 MPa (sponges/gels)	~0.05–2 MPa	Load-bearing suitability	[6,11,32]
	Tensile strength	~1–10 MPa (dense scaffolds)	~0.2–5 MPa	Determines suitability for tendon/orthopedic use	[6,36]
	Strain at break	20–60%	30–80% (more compliant)	More deformable but less stable	[20]
Degradation	In vitro enzymatic half-life (collagenase)	~3–10 days (crosslink-dependent up to weeks)	~1–5 days (unmodified)	Controls scaffold persistence	[7,33]
	MMP susceptibility	Lower (compact fibrils)	Higher (open fibrillar structure)	Faster remodeling in marine systems	[17,20]
	Stability in aqueous media	High–moderate	Low–moderate	Determines in vivo handling stability	[27]
Biological response	Cell adhesion efficiency	High (well-characterized integrin binding)	High–moderate (slightly variable)	Adhesion-driven early response	[34,35]
	Proliferation support	High	High (context-dependent)	Strongly stiffness-dependent	[32]
	Osteogenic/tenogenic differentiation support	Strong, validated	Comparable after modification	Depends on stiffness + crosslinking	[5,37]

Td: denaturation temperature; MMP: matrix metalloproteinase; Pro: proline; Hyp: hydroxyproline.

**Table 2:** Application-specific design strategies and clinical decision framework for collagen-based biomaterial selection in veterinary regenerative medicine.

Clinical Application	Tissue Requirements and Key Drivers	Preferred Source	Scaffold Format and Engineering Strategy	Clinical Decision Matrix: When to Prefer
Bone and Hard Tissue Repair	High mechanical stiffness Structural integrity under load Osteoconductivity	Mammalian (standard) Marine (with composite reinforcement)	Porous, dense sponges 3D-bioprinted matrices Incorporation of inorganic phases Robust chemical crosslinking	Prefer mammalian for: Critical-sized defects requiring prolonged structural support. High-load orthopedic scenarios. Prefer marine for: Scenarios requiring accelerated remodeling dynamics. Non-critical defects where doping can bridge the initial mechanical gap.
Tendinous and Ligamentous Injuries	Tensile strength Anisotropic guidance Resistance to high cyclic loading	Mammalian (highly preferred)	Electrospun fibrous mats Highly aligned structural scaffolds High-density crosslinking to withstand elevated physiological and inflammatory temperatures	Prefer mammalian for: Superior intrinsic tensile modulus and thermal stability under extreme physiological and mechanical stress (e.g., equine superficial digital flexor tendon). Note: Marine collagen currently requires extensive synthetic hybridization (e.g., PCL blends) to target this domain.
Skin Wound Healing	Elasticity and compliance High moisture retention Rapid cellular infiltration	Marine (preferred)	Hydrated hydrogels Lyophilized porous sponges Mild crosslinking to preserve bioactive motifs	Prefer marine for: Accelerated re-epithelialization and granulation. Lower immunogenic profile in chronic/geriatric wounds. Superior exudate management and compliance in irregular anatomical sites.
Ophthalmic/ Corneal Repair	High optical transparency Low fibril density Non-invasive application	Marine (preferred)	Ultra-thin transparent membranes In situ gelling fluids Controlled, non-toxic crosslinking to maintain light transmission while preventing rapid melting	Prefer marine for: Favorable light transmission due to smaller fibrillar diameter. Excellent biomimicry for corneal stroma replacement. Minimally invasive alternative to conjunctival grafts, avoiding general anesthesia risks in high-risk patients.
Periodontal and Oral Regeneration	Hard–soft tissue interface integration Space maintenance Localized bioactivity	Mammalian and Marine (equally viable via hybrid systems)	Multi-layered/bifunctional scaffolds Composite hydrogels filling alveolar pockets Targeted delivery of growth factors or antimicrobials	Prefer mammalian for: Guided tissue regeneration membranes requiring long-term barrier function. Prefer marine for: Injectable formulations targeting deep periodontal pockets, eliminating the need for invasive flap surgeries.

PCL: polycaprolactone.

## Data Availability

No new data were created or analyzed in this study. Data sharing is not applicable to this article.

## Supplementary Materials

The following supporting information can be downloaded at https://www.mdpi.com/article/10.3390/ani16162529/s1. Table S1. Overview of the studies included in the review. Studies are categorized by design, collagen source, scaffold format, target application, and experimental model. Principal outcomes and their relevance to the comparison between mammalian and marine collagen are summarized.
