# Supplementary material for: Collagen Scaffolds in Veterinary Medicine: Safety, Sustainability, and Clinical Accessibility of Mammalian- and Marine-Derived Sources—A Scoping Review
Source: Animals (Basel). 2026 Aug 13;16(16):2529. doi: 10.3390/ani16162529 (PMC13508881; doi:10.3390/ani16162529)
Supplement: Supplementary file 1 [file animals-16-02529-s001.zip › animals-4470430-supplementary.pdf]

animals

MDPI

Table S1. Overview of the studies included in the review. Studies are categorized by design, collagen source, scaffold format, target application, and experimental model. Principal outcomes and their relevance to the comparison between mammalian and marine collagen are summarized.

Author(s) & Year	Study design	Collagen source	Scaffold format	Target application	Animal model/species	Principal outcomes	Relevance to mammalian vs marine comparison
Badylak 2007 [1]	Review	Mammalian	N/A	ECM scaffolds	N/A	Reviews the biological activity, degradation and remodeling of ECM-derived scaffolds.	Provides a mammalian-collagen/ECM framework for scaffold function and remodeling.
Lim et al. 2019 [2]	Review	Marine	N/A	Biomedical applications	N/A	Reviews marine collagen properties, processing and biomedical applications.	Provides background on marine collagen as an alternative to mammalian sources.
Shaik et al. 2024 [3]	Review	Marine	N/A	Biomedical applications	N/A	Reviews marine collagen extraction, physicochemical properties and bioactive characteristics.	Informs assessment of processing-dependent properties of marine collagen.
Gelse et al. 2003 [4]	Review	N/A	N/A	General biomedical applications	N/A	Reviews collagen structure, biosynthesis and biological functions.	Provides the molecular framework for interpreting collagen properties across sources.
Lynn & Yannas 2004 [5]	Review	Mammalian	N/A	Collagen biomaterials	N/A	Reviews antigenicity and immunogenicity of collagen biomaterials.	Provides background for comparing biological safety considerations between collagen sources.
Silva et al. 2014 [6]	Review	Marine	N/A	Regenerative medicine	N/A	Reviews marine collagen characteristics, applications and sustainability aspects.	Provides background on marine collagen as an alternative source, including sustainability considerations.
Ricard-Blum 2011 [7]	Review	N/A	N/A	General biomedical applications	N/A	Reviews collagen structure, organization, biological functions and tissue distribution.	Provides structural and biological background for collagen biomaterials.
Coppola et al. 2020 [8]	Review	Marine	Membranes, sponges, hydrogels, films	Tissue engineering and regenerative medicine	N/A	Reviews marine collagen sources, processing and biomedical applications, including	Provides background for comparison of marine collagen with established collagen biomaterials.

			and composites			biocompatibility, degradation and thermal stability.	
Almeida et al. 2025 [9]	Review	Marine	N/A	Biomedical applications	N/A	Reviews emerging marine invertebrate collagen sources and their biomedical applications.	Expands the marine collagen evidence base beyond fish-derived sources.
Zheng et al. 2023 [10]	Review	N/A	N/A	Regenerative medicine	N/A	Reviews collagen-based biomaterial structures, crosslinking, fabrication and applications.	Provides context for assessing the influence of processing and scaffold design on collagen performance.
Raftery et al. 2017 [11]	In vitro + in vivo	Mammalian	Collagen and collagen–hydroxyapatite scaffold	Bone regeneration	Rat	Gene-activated collagen–hydroxyapatite scaffolds enhanced osteogenesis and bone formation.	Provides a mammalian-collagen benchmark for scaffold performance in bone regeneration.
Barrett et al. 2019 [12]	In vitro	Mammalian	Membrane	General tissue engineering	N/A	Developed and characterized collagen membranes using pulsed electrophoretic deposition.	Provides evidence on processing and fabrication of mammalian collagen membranes.
Felician et al. 2018 [13]	Review	Marine	N/A	Biomedical applications	N/A	Reviews marine collagen sources, extraction methods, properties and applications.	Provides background for evaluating marine collagen as an alternative collagen source.
Tricco et al. 2018 [14]	Methodological guideline	N/A	N/A	Scoping review methodology	N/A	Provides guidance for conducting and reporting scoping reviews.	Provides the methodological framework for evidence mapping in the present review.
Shoulders & Raines 2009 [15]	Review	N/A	N/A	General biomedical applications	N/A	Reviews molecular determinants of collagen structure and stability.	Provides the structural basis for interpreting collagen stability.
Fratzl 2008 [16]	Review	N/A	N/A	General biomedical applications	N/A	Reviews hierarchical collagen organization and its relationship with mechanical properties.	Provides a framework for interpreting structural and mechanical properties of collagen.
Zeugolis et al. 2008 [17]	In vitro	Mammalian	Extruded collagen fibers	Tissue engineering	N/A	Post-assembly processing altered the structural, mechanical and thermal properties of collagen fibers.	Supports consideration of processing as a determinant of collagen biomaterial performance.
Zeugolis et al. 2009 [18]	In vitro	Mammalian	Extruded collagen fibers	Tendon/ligament tissue engineering	N/A	Chemical, physical and biological crosslinking altered collagen fiber properties.	Provides a mammalian benchmark for evaluating crosslinking

							strategies used to modify collagen stability and mechanics.
Silvipriya et al. 2015 [19]	Review	Comparative (mammalian vs marine)	N/A	General biomedical applications	N/A	Reviews collagen sources, extraction, characteristics and biomedical applications.	Supports comparison of mammalian and marine collagen sources.
Liu et al. 2019 [20]	Review	N/A	N/A	General biomedical applications	N/A	Reviews collagen hierarchical structure, modification strategies and biomedical applications.	Provides context for assessing relationships between structure, processing and performance.
Yuan et al. 2026 [21]	Review	Marine	N/A	Regenerative medicine	N/A	Reviews marine collagen structure–function relationships, properties, applications and translational challenges.	Provides recent context for marine collagen properties and challenges relative to established collagen biomaterials.
Huang et al. 2016 [22]	In vitro	Marine	N/A	Physicochemical characterization	N/A	Characterized tilapia scale collagen following extrusion-assisted extraction.	Provides primary data on the physicochemical properties and processing of fish collagen.
Singh et al. 2011 [23]	In vitro	Marine	N/A	Physicochemical characterization	N/A	Characterized fish type I collagen, including thermal stability, solubility and structural properties.	Provides primary data relevant to comparison of marine and mammalian collagen stability.
Subhan et al. 2015 [24]	Review	Marine	Porous scaffolds, hydrogels, composites and fibrillar gels	Tissue engineering and regenerative medicine	Rat; in vitro human cells	Reviews marine collagen safety, thermal stability and biomedical applications, including strategies to improve stability.	Directly informs comparison of marine and mammalian collagen regarding safety, thermal stability and processing.
Sionkowska 2011 [25]	Review	N/A	N/A	Biomaterials	N/A	Reviews blending of natural and synthetic polymers to modify biomaterial properties.	Provides background for composite strategies that may address mechanical limitations of collagen scaffolds.
Nagai & Suzuki 2000 [26]	In vitro	Marine	N/A	Characterization	N/A	Fish collagen showed lower thermal stability than mammalian collagen, with denaturation temperatures around 25–30 °C.	Provides foundational evidence for the lower thermal stability of marine collagen.
Jongjareonrak et al. 2005 [27]	In vitro	Marine	N/A	Characterization	N/A	Characterized fish collagen and reported differences in thermal stability, solubility and structural	Provides evidence of source- and extraction-dependent variability in marine collagen properties.

						properties between collagen preparations.	
Orgel et al. 2006 [28]	In vitro	Mammalian	N/A	Structural characterization	N/A	Characterized the highly organized microfibrillar architecture of mammalian collagen.	Provides a structural benchmark for interpreting mechanical differences between mammalian and marine collagen.
Song et al. 2025 [29]	In vitro	Comparative (mammalian vs marine)	Hydrogel / fibrillar collagen	Biomaterials	N/A	Both collagens showed good cytocompatibility, whereas fish collagen had lower thermal stability and faster enzymatic degradation.	Provides direct comparative evidence on thermal stability and degradation of mammalian and marine collagen.
Eekhoff & Soslowsky 2026 [30]	In vitro	Mammalian	N/A	Structural characterization	N/A	Statistical nonparametric mapping enabled quantitative comparison of collagen fibril diameter distributions.	Provides a methodological basis for quantitative comparison of collagen fibrillar architecture.
Olde Damink et al. 1996 [31]	In vitro	Mammalian	Membrane	General tissue engineering	N/A	EDC/NHS crosslinking increased the thermal stability of dermal sheep collagen and altered its mechanical properties.	Provides a mammalian benchmark for crosslinking as a strategy to modify collagen stability and mechanics.
O'Brien 2011 [32]	Review	N/A	N/A	Tissue engineering	N/A	Reviews scaffold requirements including biocompatibility, porosity and mechanical integrity.	Provides general criteria for evaluating collagen scaffold suitability across sources.
Fields 2013 [33]	Review	N/A	N/A	Degradation biology	N/A	Reviews enzymatic collagen degradation and its role in tissue remodeling.	Provides the biological framework for interpreting differences in collagen degradation between sources.
Hynes 2009 [34]	Review	N/A	N/A	Cell–matrix interactions	N/A	Reviews ECM regulation of cell adhesion, signaling and behavior.	Provides the biological framework for comparing cellular responses to collagen-based biomaterials.
Leitinger 2011 [35]	Review	N/A	N/A	Cell–matrix interactions	N/A	Reviews collagen receptors and their roles in cell adhesion, migration and signaling.	Provides mechanistic context for source-dependent differences in cellular responses.
Chhaya et al. 2022 [36]	In vitro	Comparative	Membrane	Guided tissue regeneration	N/A	Bovine membranes showed greater mechanical strength and	Provides direct comparative evidence on mechanical properties and degradation.

		(mammalian vs marine)				structural organization, whereas fish membranes degraded faster.	
Ferreira et al. 2012 [37]	Review	Mammalian	Composite scaffold	Bone regeneration	N/A	Reviews collagen-based scaffolds for biomimetic bone tissue engineering and emphasizes structural and mechanical requirements.	Provides a mammalian-collagen benchmark for bone scaffold design.
Dowling et al. 2000 [38]	Review/clinical	Mammalian	N/A	Tendon repair	Horse	Reviews collagen-related pathology and limitations of tendon healing.	Provides veterinary context for collagen-dependent repair in a load-bearing tissue.
Thorpe et al. 2010 [39]	Ex vivo	Mammalian	N/A	Tendon biomechanics	Horse	Tendon mechanical strength was associated with collagen cross-linking and structural organization.	Provides a mammalian tissue benchmark for collagen-dependent mechanical performance.
Diogo et al. 2024 [40]	In vivo	Comparative (mammalian vs marine)	3D composite scaffold	Bone regeneration	Rabbit	Marine- and bovine-based composite scaffolds showed different porosity, mechanical, degradation and tissue-formation outcomes.	Provides direct comparative evidence on marine and mammalian collagen in a bone regeneration model.
Amini et al. 2012 [41]	Review	N/A	N/A	Bone tissue engineering	N/A	Reviews the combined roles of scaffolds, cells and signaling factors in bone regeneration.	Provides general context for evaluating collagen scaffold performance in bone applications.
Han et al. 2024 [42]	Review	N/A	Composite scaffold	Bone regeneration	N/A	Reviews collagen–hydroxyapatite composites and their effects on cell adhesion and osteogenesis.	Provides context for composite strategies applicable to both collagen sources.
Atlas of Small Animal Wound Management 2018 [43]	Textbook	N/A	N/A	Wound healing	N/A	Describes the biological phases of wound healing and the role of ECM in tissue repair.	Provides the biological framework for interpreting collagen scaffold performance in wound healing.
Carolo et al. 2023 [44]	In vivo	Comparative (mammalian vs marine)	Bi-layered dermal template	Skin wound healing	Rat	Marine-derived and bovine-derived dermal templates supported wound healing, with differences in early closure and angiogenesis.	Provides direct comparative evidence on marine and mammalian collagen in a wound healing model.

Zivelonghi et al. 2024 [45]	Ex vivo	Marine	Sponge / wound dressing	Wound healing	Rat skin explants	Marine collagen dressings preserved epidermal integrity and supported tissue repair; functionalization further modified the response.	Provides evidence for marine by-product collagen in wound-healing biomaterials and its potential for functionalization.
Loor-Morales et al. 2025 [46]	In vivo	Marine	Scaffold / xenograft	Wound healing	Dog	Marine collagen treatment was associated with accelerated wound closure and improved collagen organization.	Provides veterinary evidence on marine collagen in soft-tissue repair.
Melotti et al. 2021 [47]	In vivo	Marine	Skin substitute	Wound healing	Sheep	Marine-derived treatment enhanced re-epithelialization, angiogenesis and dermal maturation.	Provides large-animal evidence on marine collagen performance in skin regeneration.
Boso et al. 2020 [48]	Review	Mammalian	Hydrogel	Skeletal muscle regeneration	N/A	Reviews ECM-derived hydrogels and their effects on myoblast behavior in muscle tissue engineering.	Provides a mammalian-collagen benchmark for muscle scaffold design and cell–matrix interactions.
Akyürek et al. 2026 [49]	In vitro	Marine	3D scaffold	Skeletal muscle tissue engineering	N/A	Sea urchin collagen scaffolds supported cell viability and myogenic marker expression.	Provides emerging evidence for marine collagen in skeletal muscle tissue engineering.
Liu et al. 2020 [50]	In vitro	Mammalian	N/A	Skeletal muscle regeneration	N/A	Type I collagen promoted C2C12 migration and myogenic differentiation through IL-6/FAK/NF-κB signaling.	Provides a mammalian-collagen benchmark for cell–collagen interactions in muscle regeneration.
Li & Wang 2024 [51]	Review	N/A	Hydrogels, films and scaffolds	Corneal regeneration	N/A	Reviews collagen-based biomaterials for corneal repair and regeneration.	Provides comparative background on collagen biomaterials for ophthalmic applications.
Mobaraki et al. 2019 [52]	Review	N/A	Membrane / hydrogel	Corneal repair and regeneration	N/A	Reviews the importance of collagen organization and biomaterial properties in corneal regeneration.	Provides context for evaluating collagen source in applications requiring specific structural and optical properties.
Fagerholm et al. 2010 [53]	Clinical trial	Mammalian	Implant	Corneal regeneration	Human	Biosynthetic collagen implants supported tissue regeneration and visual improvement without immunosuppression.	Provides a clinical benchmark for collagen-based regenerative implants.

Johnston 1997 [54]	Review/clinical	N/A	N/A	Osteoarthritis	Dog	Reviews cartilage structure and ECM degradation associated with osteoarthritis.	Provides veterinary biological context for collagen-related joint disease but no direct source comparison.
Niemiec 2008 [55]	Review	N/A	N/A	Periodontal disease	Dog and cat	Reviews periodontal disease progression and tissue destruction.	Provides veterinary clinical context for periodontal regeneration but no direct collagen-source comparison.
Kosen et al. 2012 [56]	In vivo	Mammalian	Composite scaffold (hydrogel + sponge)	Periodontal regeneration	Dog	The collagen scaffold supported periodontal regeneration, including new bone, cementum and periodontal ligament formation.	Provides a canine mammalian-collagen benchmark for periodontal scaffold performance.
Sculean et al. 2015 [57]	Systematic review	Mammalian	Membranes, graft composites and combination therapies	Periodontal regeneration	Human	Collagen-based biomaterials used with surgery improved regenerative outcomes, although results varied among studies.	Provides clinical evidence supporting established mammalian collagen biomaterials and contextualizes outcome variability.
Ward 2022 [58]	Narrative review	N/A	Hydrogels, sponges, membranes and composites	Periodontal tissue engineering	N/A	Reviews scaffold-, cell-, growth factor- and gene-based approaches for periodontal regeneration.	Provides veterinary context for scaffold-based regeneration without source-specific comparison.
Wang et al. 2023 [59]	Review	N/A	Sponges, membranes, gels and composites	Broad tissue engineering	N/A	Reviews collagen biomaterials and strategies used to improve mechanical properties and degradation.	Supports assessment of processing and composite strategies across mammalian and marine collagen.
Hutmacher 2000 [60]	Review	N/A	N/A	Bone and cartilage tissue engineering	N/A	Defines scaffold design requirements including porosity, interconnectivity, biodegradation and mechanical properties.	Provides general criteria for assessing the functional suitability of collagen scaffolds from different sources.
Pal & Suresh 2016 [61]	Review	Marine	N/A	Biomaterials and other applications	N/A	Reviews seafood by-products as collagen sources and discusses extraction, processing and commercialization challenges.	Provides evidence relevant to the sustainability, accessibility and processing considerations of marine collagen sources.

Murphy & Atala 2014 [62]	Narrative review	N/A	3D-printed scaffolds and bioinks	Tissue and organ engineering	N/A	Reviews 3D bioprinting strategies using natural and synthetic biomaterials, including collagen-based systems.	Provides general context on collagen as an ECM-mimicking biomaterial but does not address source-specific differences.

Abbreviations: N/A, not applicable or not reported in the original study.
Collagen source was classified as mammalian, marine, comparative (mammalian vs marine), or not specified, based strictly on explicit information reported in each study, without inference when not clearly stated.
